# Thermal Evolution of Scallop and Surf Clam Shell: Phase Transformation Insights from Solid-State ^13^C NMR and Multi-Technique Microanalysis

**DOI:** 10.3390/ma19143040

**Published:** 2026-07-14

**Authors:** Novik Kurohman, Heesup Choi, Masumi Inoue, Masato Kida

**Affiliations:** 1 Graduate School of Engineering, Kitami Institute of Technology, Kitami 090-8507, Hokkaido, Japan; d3247180043@std.kitami-it.ac.jp; 2 Division of Civil and Environmental Engineering, Kitami Institute of Technology, Kitami 090-8507, Hokkaido, Japan; m-inoue@mail.kitami-it.ac.jp (M.I.); mkida@mail.kitami-it.ac.jp (M.K.)

**Keywords:** seashell biowaste, CaCO_3_, calcination, cement replacement, solid-state ^13^C NMR

## Abstract

Globally, large quantities of discarded shells, including scallops and surf clams, are often accumulated at disposal sites, causing environmental degradation. Calcium carbonate (CaCO_3_) in seashells predominantly exists in polymorphic forms, such as aragonite and calcite, and undergoes phase transformation upon thermal treatment. This study investigates the thermal evolution of scallops and surf clam shells at various calcination temperatures to characterize changes in crystalline structure, pore morphology, and to review their potential as partial cement replacement materials. The seashells were mechanically ground into fine powder and subsequently calcined at 650 °C, 750 °C, and 850 °C for 7 h. The CaCO_3_ transformation was comprehensively characterized using solid-state ^13^C NMR, XRD, TG-DTA, and SEM to resolve carbonate speciation, phase transitions, decomposition thermodynamics, and microstructural evolution. The solid-state ^13^C NMR was employed to analyze the local chemical environments of carbonate species and to provide additional spectroscopic evidence supporting the phase identification obtained from XRD and TG-DTA. Furthermore, XRD, TG-DTA, and SEM analyses consistently confirmed the phase transformations and associated changes in pore structure and crystallinity induced by calcination. These findings suggest that solid-state ^13^C NMR can be a useful technique for tracking CaCO_3_ decomposition, while calcination may enhance the potential of seashell biowaste as a sustainable cementitious material.

## 1. Introduction

**Figure 1:**
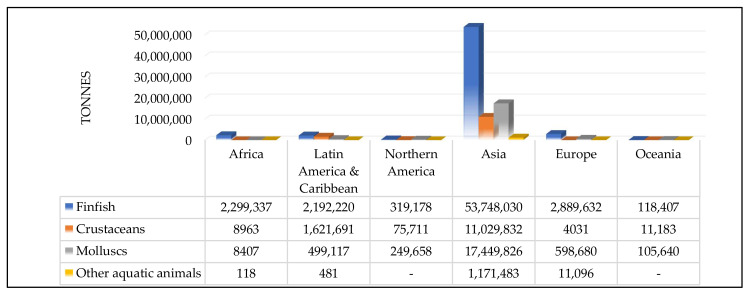
Regional distribution of global inland and marine/coastal aquaculture production by main species categories, 2022 [5].

**Figure 2:**
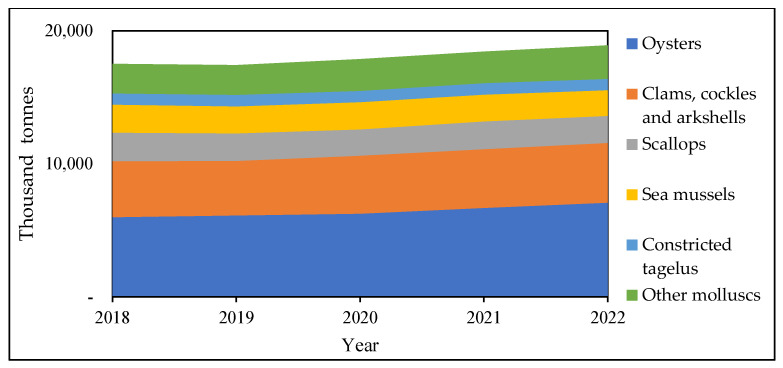
Global aquaculture production of major molluscs species (oysters, mussels, clams, and scallops) based on FAO 2022 statistics [5].

**Figure 3:**
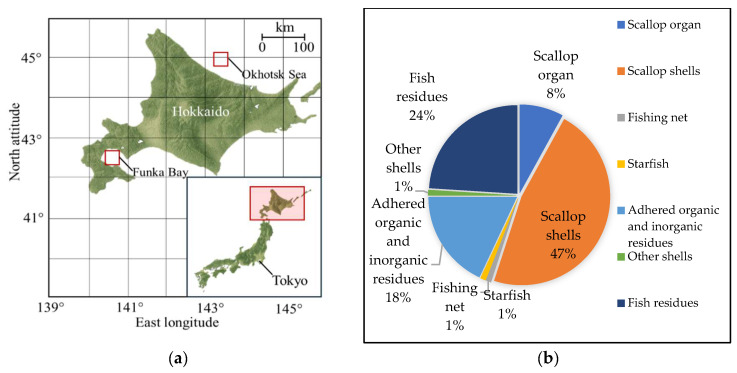
Scallop harvesting regions in Hokkaido (**a**) and the composition of marine waste in Hokkaido, Japan, 2024 (**b**) [17].

Southeast Asia generates around 1.5 million tons of seashell waste per year, while worldwide production of marine waste, including crab, shrimp, and lobster, reaches an estimated 6–8 million metric tons annually [1,2]. Figure 1 and Figure 2 show the global distribution and production trend of aquaculture species. Asia dominated global aquaculture production, particularly for finfish, molluscs, and crustaceans. In addition, global mollusc species production gradually increased from 2018 to 2022, with oysters showing the highest production, followed by clams and scallops. In Japan, annual scallop production is approximately 500,000 tons, with an estimated economic value of 84 billion JPY (about 840 million USD) [3]. As illustrated in Figure 3, nearly 60% of this seashell waste originates from Hokkaido, with notable contributions from the Okhotsk Sea and Funka Bay regions [4]. Mostly, seashell waste is directly discarded into landfills, rivers, or oceans, leading to serious environmental and public health issues.

Landfill disposal of scallop shells may pose environmental concerns, including potential soil contamination and malodorous emissions [4]. Seashells are primarily composed of CaCO_3_ in the crystalline forms of calcite and aragonite [6]. Moreover, these materials exhibit distinct thermal and mechanical properties [7]. Extensive research has explored the valorization of seashell waste for application across multiple sectors. In agriculture, seashells have been widely used as amendments to correct soil acidity and as adsorbents for the remediation of toxic metal-contaminated wastewater [8]. In medical and industrial fields, seashell-derived materials can be repurposed to synthesize nano-hydroxyapatite, which is widely used in biomedical applications, such as bone implants [9]. In the construction sector, seashell powders have attracted growing interest as partial replacements and supplementary filler materials [10,11,12,13]. The incorporation of seashell biowaste into cementitious materials offers a potentially viable approach to partial waste valorization, potentially reducing marine biowaste accumulation and alleviating associated disposal challenges.

Various seashell species, such as scallops and surf clams, may differ in chemical composition, which can influence their responses to thermal treatment. Seashells are composed of approximately 95% CaCO_3_, with the remaining fraction consisting of organic matter and minor impurities [14]. Calcite, one of the principal polymorphs of CaCO_3_, is thermodynamically stable under ambient conditions and demonstrates greater thermal stability compared to aragonite over a wide range of temperatures [15]. In contrast, aragonite readily transforms into calcite at elevated temperatures and under mechanical stress [16]. Thermal processing is therefore used to decompose and remove organic matter embedded in the shell matrix, thereby increasing CaO purity and improving mechanical performance. Thermal treatment is applied to remove residual organic constituents from the shell matrix and to promote the formation of high-purity CaO, thereby enhancing its reactivity and contribution to mechanical performance. Furthermore, calcination increases the surface area and induces significant changes in the material’s structural, morphological, and crystalline characteristics [16].

Morphologically, the scallop shell has a typical fan shape with distinct radial ribs, giving it a ribbed external surface. It exhibits a lamellar structure, which contributes to its mechanical strength and structural durability in a marine environment [18]. This layered arrangement consists of alternating CaCO_3_ layers and organic protein matrices, forming a structure that is both strong and lightweight, which may contribute to its favorable mechanical properties [19,20]. CaCO_3_ obtained from seashells plays a crucial role in cementitious systems, improving mechanical performance, hydration behavior, compressive and tensile strength, workability, and porosity [20,21]. When calcined at approximately 800 °C, seashell-derived calcium carbonate decomposes into calcium oxide (CaO), which can serve as a partial replacement for Portland cement, thereby reducing the carbon footprint associated with cement production [22]. CaO may rapidly hydrate during curing to form Ca(OH)_2_, which subsequently facilitates pozzolanic interactions with silica-rich constituents, thereby accelerating hydration kinetics and improving early-age strength development [23]. Moreover, previous studies have reported that appropriate CaO contents can accelerate the formation of calcium silicate hydrate (C–S–H) gel and other hydration products, thereby enhancing hydration kinetics and early-age strength development. In addition, reactive CaO can promote rapid hydration and contribute to the formation of ettringite and other hydration products that are essential for strength development in cementitious materials [23]. The incorporation of calcined seashell-derived materials has also been reported to refine the pore structure, reduce porosity, and improve the compactness of the cement matrix [24]. However, excessive free CaO may cause expansion or shrinkage, which can reduce compressive strength and long-term durability. Therefore, careful control of the calcination process and appropriate dosing of calcined shell-derived materials are important considerations for their potential as a source of reactive CaO in future investigations. Maintaining a substitution ratio of approximately 5% can help preserve concrete’s mechanical properties [25]. Despite these advancements, the mechanisms underlying thermal evolution and atomic-scale transformations during the calcination of seashell-derived CaCO_3_ remain insufficiently explored. While techniques such as XRD and FT-IR are effective for identifying phase transitions and functional group changes, they provide less direct information on the local chemical environments of specific nuclei, particularly carbonate species (CO_3_^2−^), during decomposition.

NMR spectroscopy has emerged as an indispensable analytical technique for the advanced characterization of cementitious materials. The inherent chemical complexity of cement systems, encompassing a multitude of crystallographically disordered phases that continuously evolve after mixing, poses considerable challenges to elucidating their atomic-scale structural configurations. Fundamentally, NMR probes the interactions of nuclei with intrinsic magnetic moments with an external magnetic field, providing information about their local chemical environments [26]. In cementitious systems, a range of nuclei, including ^1^H, ^13^C, ^17^O, ^19^F, ^23^Na, ^25^Mg, ^27^Al, ^29^Si, ^31^P, ^33^S, ^35^Cl, ^39^K, and ^43^Ca, are commonly investigated to elucidate atomic structure, phase evolution, nanostructure development, and reaction mechanism [26]. NMR methods, including ^13^C MAS and ^13^C cross-polarization (CP) NMR, are highly effective in elucidating the mechanisms and extent of carbonation in cementitious materials. For instance, ^13^C Magic Angle Spinning (MAS) NMR spectroscopy has been employed as a quantitative analytical approach for the identification and quantification of carbonate phases in cementitious systems, while ^13^C cross-polarization (CP) NMR plays an important role in identifying hydrous carbonate species in these materials [27,28]. Beyond carbon characterization, NMR techniques have been applied widely to investigate guest ions in principal cement phases such as aluminum, phosphorus, and fluorine in alite and belite, as well as aluminum and zinc in C-(A)-S-H structures, enabling the differentiation between hydrous and anhydrous carbonate environments [27,29,30,31,32,33]. Thaumasite (Ca_6_Si(OH)_6_(CO_3_)_2_(SO_4_)_2_·24H_2_O) forms in concrete through the interaction of calcium silicate hydrate (C-S-H), carbonate, and sulfate ions, and its presence can be identified and characterized by using NMR analyses [29]. Moreover, solid-state NMR spectroscopy (^1^H and ^13^C) has been applied to investigate various calcium carbonate phases, including calcite, aragonite, vaterite, monohydrocalcite (CaCO_3_·H_2_O), and ikaite (CaCO_3_·6H_2_O) [34]. Notably, this technique allows reliable differentiation between CaCO_3_ polymorphs and pseudopolymorphs, as variations in the local chemical environments of carbonate ions produce distinct ^13^C NMR chemical shifts [34,35].

Although ^13^C MAS NMR has been extensively utilized to examine carbonate species and carbonation mechanisms, its application in monitoring the phase transition behavior of CaCO_3_ during thermal decomposition remains relatively limited. In this study, the transformation of CaCO_3_ derived from scallop and surf clam shell waste during the calcination process is monitored using single-pulse ^13^C MAS NMR spectroscopy. This technique enables direct observation of ^13^C nuclei without polarization transfer from other nuclei, providing a straightforward yet reliable approach for detecting structural transitions [36,37]. Moreover, solid-state ^13^C NMR is particularly useful for distinguishing different carbon environments within CaCO_3_, such as carbonate (CO_3_^2−^) from bicarbonate (HCO_3_^−^) [38]. The results may provide important insights into the decomposition behavior of CaCO_3_ and demonstrate that integrating solid-state ^13^C NMR with microanalytical techniques, including XRD, TG-DTA, and SEM, offers an effective approach for monitoring these transformations. Furthermore, the findings suggest that calcination may enhance the viability of seashell biowaste as a sustainable cementitious material in future investigations.

## 2. Materials and Methods

### 2.1. Materials

**Figure 4:**
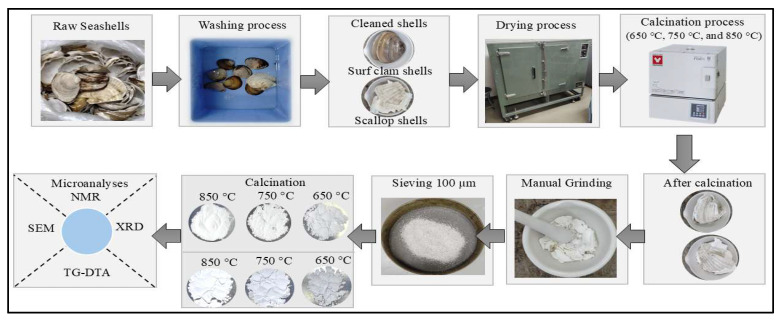
Experimental workflow for thermal treatment and microanalysis.

**Figure 5:**
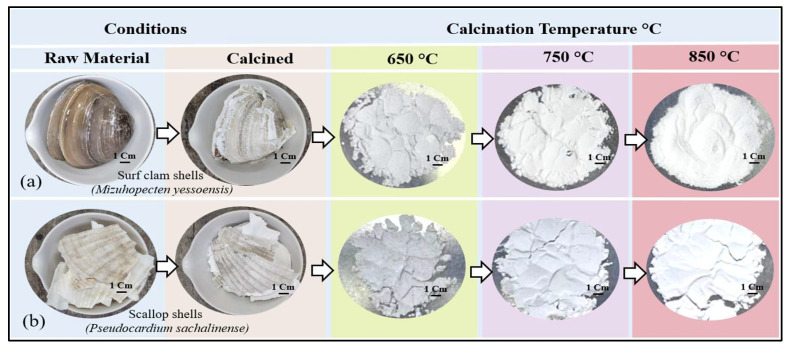
Optical images of the (**a**) surf clam and (**b**) scallop shell with and without calcination at 650 °C, 750 °C, and 850 °C for 7 h.

The waste scallop and surf clam shells used in this study were collected from Kitami, Japan. These species were selected due to their widespread availability and abundance in the local area. The harvested shell specimens were subjected to an initial washing procedure to remove surface-bound contaminants, including sand, soluble salts, and organic residues. For the preparation of calcined specimens, the pre-cleaned shells were thermally dried in a laboratory oven at 105 °C for 24 h to eliminate residual moisture and eradicate microorganisms on the surface and within the internal pore structure. The dried shells were subsequently calcined in an electric furnace (FO-201, Yamato, Tokyo, Japan). The samples were heated from room temperature to the target temperatures (650 °C, 750 °C, and 850 °C) at a heating rate of 16 °C min^−1^, then held at each target temperature for 7 h to capture the progressive stages of thermal decomposition, as illustrated in Figure 4 and Figure 5 The calcination duration was selected based on previous studies of biogenic calcium carbonate materials, which demonstrated that prolonged heating may promote the decomposition of CaCO_3_ and the formation of CaO [39]. After calcination, the samples were pulverized using a ceramic grinder and subsequently passed through a 100 µm sieve to ensure a consistent particle size distribution. For comparison, the uncalcined shells were similarly cleaned, dried, ground, and then sieved through the same 100 µm mesh to ensure consistency in particle size. Both calcined and uncalcined powders were then stored in a vacuum chamber to prevent moisture absorption and maintain their purity. The thermal transition of CaCO_3_ to CaO is illustrated by the following chemical equation [40].

CaCO_3_ (s) → CaO (s) + CO_2_ (g) (1)

### 2.2. Microstructural Characterization

#### 2.2.1. NMR Measurements

Solid-state ^13^C NMR spectra were obtained using a Bruker ULTRASHIELD 400 WB PLUS spectrometer (Bruker BioSpin GmbH, Rheinstetten, Germany) operating for carbon nuclei at a resonance frequency of 100 MHz. Measurements were conducted under magic-angle-spinning (MAS) conditions at 10,000 Hz using a 4 mm rotor. The ^13^C MAS NMR spectra were acquired using a 90° pulse of 5.0 μs, a recycle delay of 540 s, and 160–600 scans. All experiments were carried out under ambient atmospheric conditions. The sample temperature during MAS at 10 kHz was estimated to increase by approximately 27 °C based on the ^79^Br chemical shift calibration of KBr, following the method reported by Thurber and Tycko (2009) [41]. The solid-state ^13^C NMR technique was employed to examine the crystalline structure of the samples before and after calcination. The resulting spectra provide clear evidence of atomic-scale structural evolution and of the progressive decomposition of CaCO_3_ during calcination.

#### 2.2.2. XRD Measurements

The phase composition and crystalline structure of the samples before and after calcination were characterized using an Ultima IV X-ray diffractometer (Rigaku Corporation, Akishima, Tokyo, Japan) equipped with CuKα radiation (λ = 1.5406 Å) and operated at 40 kV and 20 mA. Diffraction patterns were recorded over a 2θ range of 5–65° with a scanning rate of 2° min^−1^. XRD analysis was used to investigate the mineralogical transformation of CaCO_3_ to CaO during thermal treatment.

#### 2.2.3. TG-DTA Measurements

The thermal decomposition behavior of the samples was examined using a TG-DTA system (Thermo Plus TG-DTA, Rigaku Corporation, Akishima, Tokyo, Japan) under a nitrogen atmosphere at a heating rate of 10 °C min^−1^. Approximately 15 mg of each sample, including both calcined and uncalcined specimens, was subjected to analysis. The resulting TG-DTA curves provided detailed information on mass loss behavior, the decomposition of CaCO_3_ into CaO, and the associated evolution of CO_2_ during heating.

#### 2.2.4. SEM Analysis

The surface characteristics of the samples were investigated using a scanning electron microscope (SEM, JSM-6510A; JEOL Ltd., Tokyo, Japan). Observations were conducted at 15 kV to obtain detailed micrographs of particle morphology. Before analysis, the samples were sputter-coated with a thin platinum (Pt) layer to improve conductivity and suppress charging effects. The SEM images were subsequently analyzed to assess morphological and microstructural changes in CaCO_3_ induced by calcination treatment.

## 3. Results

### 3.1. NMR Results

**Figure 6:**
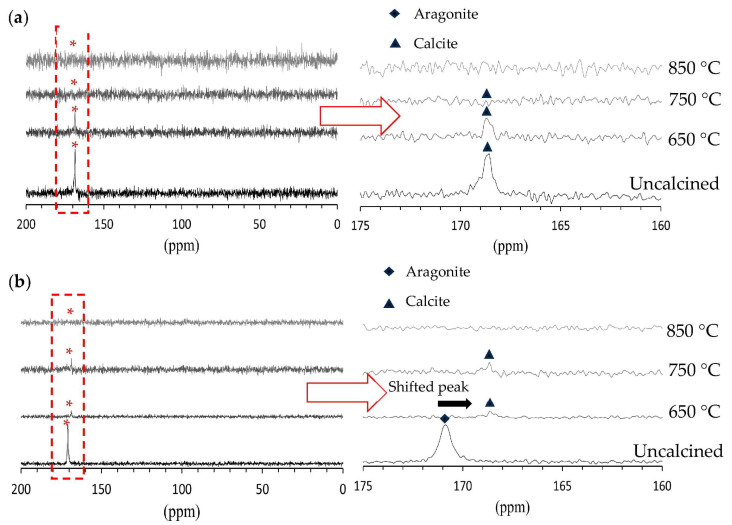
^13^C MAS NMR spectra of (**a**) scallop and (**b**) surf clam shells at different calcination temperatures. The right panels show the expanded carbonate region (160–175 ppm), indicating an aragonite–calcite transformation and the disappearance of carbonate after CaCO_3_ decomposition. The insets highlight the isotropic peak (indicated by an asterisk).

In the present study, single-pulse ^13^C MAS NMR was employed to identify the dominant CaCO_3_ polymorphs in uncalcined scallop and surf clam shells. The uncalcined scallop shell exhibited a distinct resonance at 168.7 ppm, attributed to calcite based on its characteristic chemical shift, narrow linewidth, and agreement with previously reported literature values (Table 1). In contrast, the surf clam shell showed a dominant resonance at 171.2 ppm, indicative of the presence of aragonite (Figure 6). These spectral assignments are consistent with the distinct crystallographic structures of CaCO_3_ polymorphs. Calcite crystallizes in a trigonal crystal system with a rhombohedral lattice, resulting in a relatively symmetric and energetically stable structure. By comparison, aragonite adopts an orthorhombic crystal system with lower symmetry, while vaterite is characterized by a more disordered, spherical metastable arrangement [42]. From a thermodynamic standpoint, calcite is the most stable polymorph under ambient conditions, followed by the less stable aragonite, while vaterite represents the most metastable phase among the three crystalline forms [42,43,44]. Therefore, understanding the transformation pathways among these polymorphs is essential not only for interpreting their distinct NMR signatures but also for evaluating their stability and reactivity. This knowledge is particularly important for predicting their behavior and performance in cementitious systems, where phase stability and transformation can directly influence durability and long-term material properties.

Prior studies have utilized ^13^C MAS NMR to investigate carbonate ions in CaCO_3_ polymorphs, with emphasis on understanding the structure of amorphous calcium carbonate and the possible presence of bicarbonate-related defects in the calcite lattice [34,45]. Slow-speed ^13^C MAS NMR has been shown to effectively differentiate CaCO_3_ polymorphs, including calcite and aragonite, under varying magnetic field strengths (9.39 T and 14.09 T) and spinning rates (νR = 1500 Hz and 800 Hz, respectively) [28]. Typically, the ^13^C resonance of carbonate carbon in calcite appears near 168.8 ppm, whereas aragonite and vaterite exhibit signals around 171.2 ppm and 168.7 ppm, respectively, reflecting differences in their crystallographic environments. However, NMR peak characteristics are not solely determined by crystal structure and can be influenced by several experimental and environmental factors. Peak broadening may result from factors such as temperature fluctuations, magnetic field inhomogeneity, and variations in the local chemical environment arising from surrounding ions or molecular interactions [46,47,48]. Furthermore, higher magnetic field strengths have been shown to favor the selective formation of certain crystalline phases, such as aragonite and vaterite, which may, in turn, influence the observed NMR resonances [49,50,51]. Additionally, variations in the chemical environment, such as calcium concentration [52], carbonation within calcium silicate hydrate (C-S-H) phases, and microbially induced calcium carbonate precipitation (MICP) can modify the structural and physical properties of CaCO_3_, leading to corresponding shifts or broadenings in the NMR spectra [52,53,54].

Understanding the thermal transformation of CaCO_3_ polymorphs is essential for establishing optimal calcination conditions to produce high-purity CaO from biogenic sources. In this study, the progressive attenuation and eventual disappearance of the ^13^C MAS NMR carbonate resonances as calcination temperature increases (Figure 6 and Table 2) reflect changes in the chemical environments of carbonate species during calcination. This evolution of the NMR signal serves as a sensitive indicator of structural breakdown within the carbonate lattice. Based on the spectral changes, three distinct thermal regimes can be clearly identified. The initial stage, occurring at approximately 650 °C, may correspond to the onset of lattice destabilization, during which the carbonate structure begins to weaken. The second stage, at around 750 °C, represents advanced decomposition, characterized by a substantial reduction in carbonate signal intensity, indicating significant progression of CaCO_3_ breakdown. Finally, at 850 °C, the complete disappearance of the carbonate resonance confirms full decarbonation and the transformation of CaCO_3_ into CaO.

Further, at 650 °C, scallop shells exhibit a weakened yet discernible calcite peak, reflecting incomplete decomposition of the carbonate lattice. This reduction in resonance intensity reflects the initial stages of lattice destabilization, in which thermal stress simultaneously induces bond strain and promotes the progressive release of CO_2_ during calcination of CaCO_3_ [55,56]. The surf clam shell, which is aragonite-dominated in its uncalcined state, shows a markedly different response at 650 °C. It undergoes CaCO_3_ transformation more rapidly. The aragonite peak at 171.2 ppm weakens substantially at 650 °C, indicating that aragonite may have lower thermodynamic stability than calcite [57,58]. In particular, the transformation of aragonite to calcite observed in the clam shell is governed by temperature and the intrinsic thermodynamic stability of each mineral phase. Aragonite typically converts to calcite between 420 °C and 460 °C (693 K–733 K), consistent with its lower stability under ambient conditions, which makes it more prone to thermally induced transformation and faster decomposition [57]. The mechanism of this transition involves the coordinated migration of CO_3_^2−^, specifically from aragonite to calcite, which is determined by a reconstructive phase transition, whereby the orthorhombic lattice of aragonite destabilizes and reforms into the trigonal lattice characteristic of calcite [57,59]. This rearrangement process occurs through migrations of CO_3_^2−^ triangles within the Ca layers [60].

However, the exact temperature of the aragonite-to-calcite transformation may vary depending on material characteristics and experimental conditions. In biogenic calcium carbonate systems, the transformation behavior can be influenced by residual organic matter, trace impurities, and the hierarchical shell microstructure. Previous studies have reported that sulfate ions (SO_4_^2−^) can inhibit the aragonite-to-calcite transformation, thereby facilitating the preservation of aragonite [61]. This study showed that dissolved sulfate (SO_4_^2−^) inhibits the aragonite-to-calcite transformation under hydrothermal conditions by suppressing the fracture network required for calcite replacement. In addition, structural characteristics such as crystal integrity and defect distributions, as well as external factors including pressure, pH, salinity, and other environmental conditions, may further influence the transformation process [24,39,62]. Consequently, residual aragonite domains may remain detectable at higher temperatures, contributing to the persistence of aragonite-related signals observed at 650 °C in the present study. At 750 °C, both shell types exhibit pronounced further reduction in their carbonate NMR signals. For scallop shells, the calcite resonance at 168.7 ppm undergoes a dramatic intensity loss, indicating that most of the calcite lattice has decomposed to CaO and CO_2_ by this temperature. For surf clam shells, the weak calcite resonance that emerged at 650 °C is further attenuated at 750 °C, consistent with the continued progression of CaCO_3_ decomposition. The near-complete loss of carbonate resonances at this temperature confirms that, at elevated temperatures, the residual carbonate phases may have transitioned into highly disordered environments that no longer contribute resolved ^13^C resonances [63,64]. In addition, the intermediate chemical shift observed for the residual signal at 750 °C is interpreted as arising from a superposition of residual aragonite and nascent calcite phases within an incompletely transformed matrix. Definitive spectral deconvolution of these overlapping contributions was not achievable, given the constraints of the present dataset.

Moreover, the transition to 850 °C, as shown in Figure 6, results in the near-total loss of all carbonate-related NMR signals in both shell types, indicating full decomposition and the formation of CaO, a phase where ^13^C NMR spectra are diminished due to the absence of CO_3_^2-^ functional groups. The disappearance of these resonances indicates the complete loss of CO_3_^2−^ groups and the reorganization of the crystalline structure into CaO-dominated domains, as further confirmed by the XRD results discussed in the following section. This complete loss of the isotropic transition in CaCO_3_ to CaO occurs at higher temperatures, approximately 600–800 °C [65,66,67]. The absence of any residual carbonate signals at 850 °C in the present study may confirm that calcination was sufficient to fully decompose calcite and aragonite, yielding high-purity CaO. In this research, the molecular-level insights afforded by ^13^C MAS NMR are substantially reinforced through integration with XRD, TG-DTA, and SEM analyses, which collectively interrogate complementary structural, thermal, and morphological characteristics across multiple length scales.

Phase assignments based on ^13^C MAS NMR, identifying calcite in scallop shells and aragonite in surf clam shells, show strong consistency with XRD phase analysis. As elaborated in the subsequent section, the progressive decrease in the ^13^C resonance intensity of carbonates with increasing temperature closely aligns with the diminishing intensity of characteristic carbonate diffraction peaks in XRD patterns. At elevated temperatures, the emergence of CaO reflections coincides with the complete disappearance of NMR signals, confirming that signal loss reflects true decarbonation. TG-DTA analyses further substantiate these findings by revealing temperature-dependent CO_2_ evolution consistent with CaCO_3_ decomposition. The slightly earlier signal attenuation observed for aragonite in NMR aligns with its earlier mass loss in TG curves, suggesting its lower thermodynamic stability relative to calcite. Furthermore, SEM observations in the next discussion may reveal a clear transition from dense, ordered biogenic structures in uncalcined samples to increasingly porous and fragmented morphologies after calcination, consistent with CO_2_ release and lattice breakdown. Collectively, these converging lines of evidence confirm that complete decarbonation occurs at 850 °C, yielding high-purity CaO with a porous microstructure.

### 3.2. XRD Results

**Figure 7:**
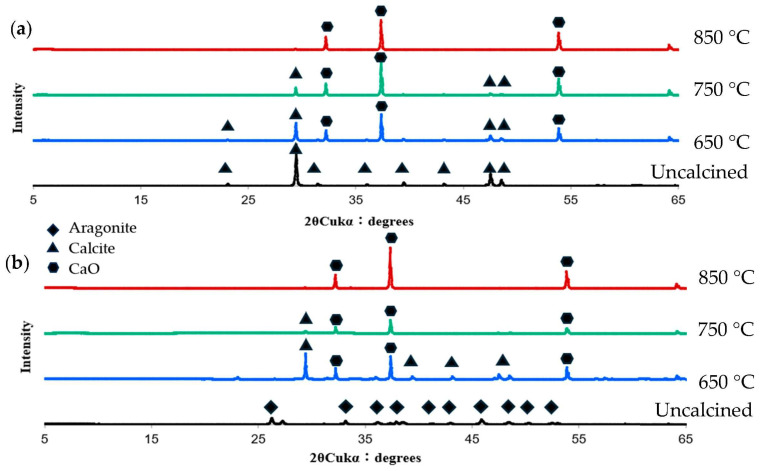
The comparison between XRD spectra of scallop shells (**a**) and surf clam shells (**b**) (before and after calcination).

CaCO_3_ precipitation may produce three anhydrous polymorphs (calcite, vaterite, and aragonite), in addition to hydrated phases such as monohydrocalcite and the hexahydrate ikaite, as well as an amorphous phase under aqueous conditions [68,69,70]. Morphologically, CaCO_3_ crystals are typically observed as cubic forms for calcite, spherical structures for vaterite, and needle-like morphologies for aragonite [70,71,72]. In this study, XRD was conducted to verify the transformation of CaCO_3_ into CaO during calcination and to identify the resulting mineral phases. The qualitative mineralogical analysis revealed a distinct polymorphic difference between the two biowaste samples, both before and after calcination. The board diffraction peak patterns of the uncalcined scallop shell in Figure 7a showed diffraction angles (2θ) at 23.1°, 29.48°, 36.0°, 47.5°, and 48.5°, corresponding to the dominant calcite peak. This confirms that calcite is the primary carbonate phase present naturally in scallop shells. As the calcination temperature increased from 650 °C to 850 °C, the calcite peak progressively weakened and eventually disappeared, while new peaks corresponding to CaO appeared. When scallop shells are subjected to heat treatment at temperatures > 800 °C, CaCO_3_ undergoes thermal decomposition and is transformed into CaO through an endothermic reaction [73], as expressed in Equation (2). This trend indicates progressive initiation and decomposition via an endothermic decarbonation process, demonstrating that full transformation to CaO is effectively achieved at 850 °C, consistent with the solid-state ^13^C NMR result.

CaCO_3_(s)→CaO(s) + CO_2_(g) ∆H298K = 178 kJ/mol (2)

On the contrary, the uncalcined surf clam shells, Figure 7b, exhibit a mineralogical profile dominated by aragonite. Distinct diffraction reflections characteristic of the aragonite phase were observed at 2θ = 26.2°, 26.3°, 38.0°, 43.2°, and 45.9°, confirming aragonite as the predominant carbonate polymorph in the surf clam shells. Furthermore, within the temperature range of 650 °C to 750 °C, aragonite transformed into calcite and progressively disappeared before completely decomposing into CaO at 850 °C. The structural changes observed by XRD are clearly aligned with the solid-state ^13^C NMR result, which shows a gradual shift from aragonite to calcite at 650–750 °C. The conversion of CaCO_3_ to CaO creates substantial porosity from CO_2_ release, with significant potential to improve material reactivity [74]. At elevated temperatures, the CaO phase exhibits superior stability and reacts chemically with water vapor to form Ca(OH)_2_. Furthermore, prolonged calcination at higher temperatures enhances recrystallization, leading to increased particle size and decreased surface area [75].

**Figure 8:**
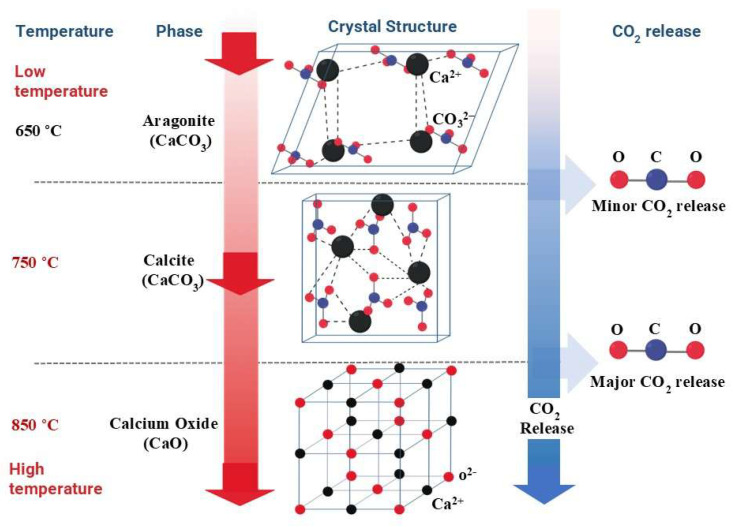
Conceptual illustration of the potential phase transitions of seashell CaCO_3_ under thermal treatment, adopted from [16,79].

The major differences observed between scallop and surf clam shells can be partly attributed to their distinct CaCO_3_ polymorphic compositions. In addition to temperature effects, this transition is influenced by factors such as residual organic content, present ions, pressure, and environmental conditions [76]. Surf clam shells are predominantly composed of aragonite, which is thermodynamically less stable and may transform into calcite upon heating before undergoing complete decarbonation to CaO [16,75,77]. Conversely, scallop shells are rich in calcite, enabling a more direct and rapid decomposition to CaO during calcination [16,78]. To gain a better understanding of this phenomenon, Figure 8 depicts a progressive phase transformation from aragonite (CaCO_3_, orthorhombic) to calcite (CaCO_3_, trigonal) induced by heat treatment. Initially, the metastable aragonite structure undergoes a structural rearrangement and begins to decompose into CaO. This thermal process proceeds in two distinct stages: a limited CO_2_ release associated with the aragonite-to-calcite transformation, followed by a substantial CO_2_ release during the decomposition of calcite into CaO. To further verify these thermal transformations, the TG-DTA results are discussed in the following section, highlighting mass loss and heat flow during calcination.

### 3.3. TG-DTA Results

**Figure 9:**
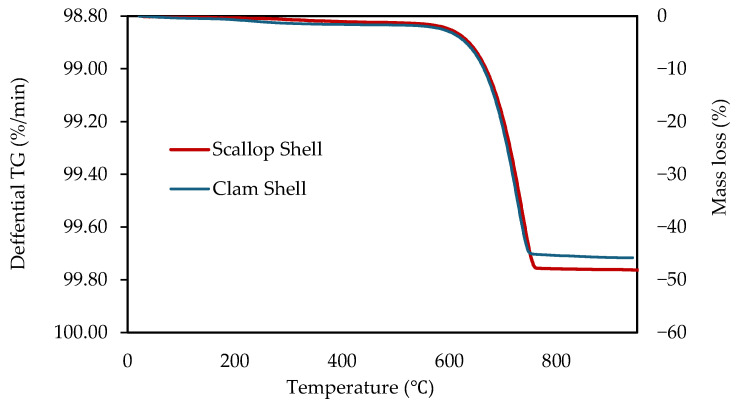
TGA results in scallop and clam shells for uncalcined samples.

**Figure 10:**
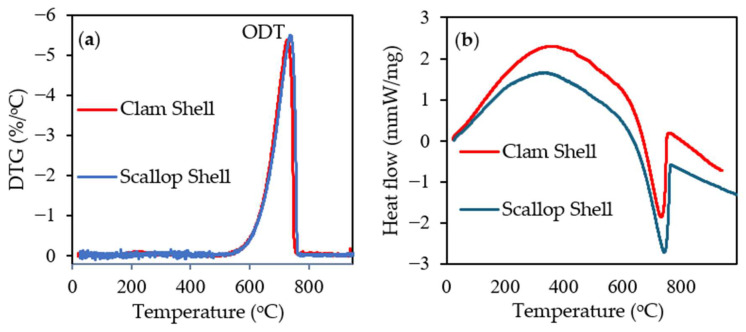
DTG results (**a**) and DTA results (**b**) for the scallop and clam shell before calcination.

The TGA results in Figure 9 show similar thermal behavior for uncalcined scallop and surf clam shells, each with three mass-loss stages (Table 3). The initial stage occurs up to 125 °C, attributed to the removal of residual adsorbed moisture [80], accompanied by a minor mass loss of approximately 0.10–0.15 wt%. The second stage, observed within the temperature range of 125–550 °C, is associated with the thermal decomposition of organic components [81]. The result of mass loss in this stage is relatively minor, resulting in a small mass loss of 1.80 wt% for scallop shells and 1.85 wt% for surf clam shells. The most significant mass loss occurs in the third stage, between 550 °C and 795 °C, corresponding to the decomposition of CaCO_3_ into CaO, accompanied by the release of CO_2_ [22]. Scallop shells show a mass loss of 46.21 wt%, whereas clam shells exhibit 43.15 wt%, corresponding to the release of CO_2_. Figure 10a also may indicate optimum degradation temperatures of approximately 740 °C for scallop shells and 730 °C for clam shells.

In agreement with these results, scallop shells initiate decomposition at approximately 550 °C and achieve complete calcination at around 770 °C, while clam shells complete decomposition at a slightly lower temperature of about 750 °C, indicating comparatively lower thermal stability. This behavior is likely related to the higher calcite content in scallop shells, given that calcite represents the most thermodynamically stable form of CaCO_3_ under ambient conditions [82,83]. In contrast, aragonite, which is dominant in clam shells, is metastable and might transform into calcite during heat treatment [82]. Additional evidence for this behavior is observed in the DTA curves in Figure 10b, where both shell types display broad endothermic peaks corresponding to the decomposition of CaCO_3_ into CaO and CO_2_ [84]. The endothermic process requires the absorption of heat to proceed, predominantly at temperatures between 600 °C and 900 °C [84,85]. The observed endothermic peak profiles suggest a gradual decomposition over a broad temperature range, in agreement with the continuous mass loss observed in the TGA curves in Figure 10.

**Figure 11:**
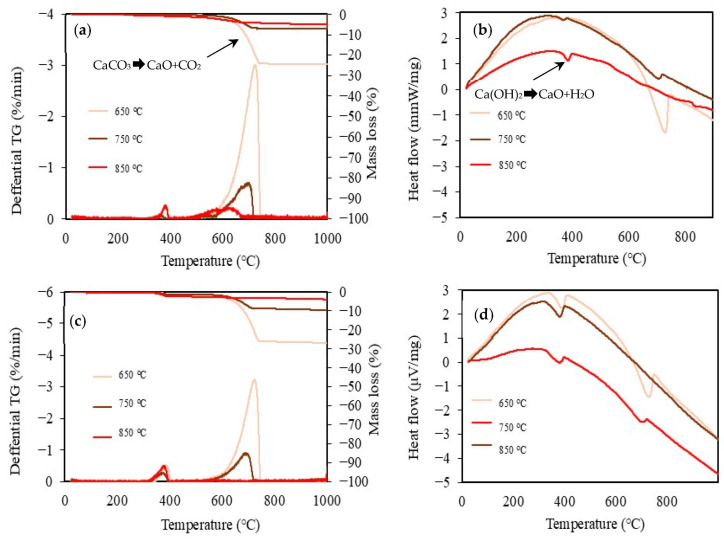
(**a**) TG-DTG scallop shell. (**b**) TG-DTA scallop shell. (**c**) TG-DTG surf clam shell. (**d**) TG-DTA surf clam shell.

Figure 11 presents the combined TG-DTG and TG-DTA curves for seashell samples after calcination at 650 °C, 750 °C, and 850 °C. Both shells reveal similar decomposition trends, characteristic of CaCO_3_ thermal dissociation. At 650 °C, partial decomposition appears, resulting in a mixture of residual CaCO_3_ and formed CaO. As the temperature increases to 750 °C, the decomposition process accelerates, reducing the remaining CaCO_3_ content and increasing CaO formation. Finally, at 850 °C, the calcination process is almost complete, as indicated by additional mass loss and the absence of CaCO_3_. At this temperature, visual observation indicated a noticeable whitening of the calcined products, consistent with extensive decomposition of CaCO_3_ as shown in Figure 5 [86,87]. These findings are in excellent agreement with the XRD and solid-state ^13^C NMR results, which consistently demonstrate the progressive transformation of CaCO_3_ to CaO at elevated temperatures.

As illustrated in Figure 11b,d, a slight mass loss occurs within the temperature range of 300–500 °C, corresponding to the thermal decomposition of calcium hydroxide (Ca(OH)_2_) into CaO and water vapor. This observation underscores CaO’s susceptibility to hydration during post-calcination handling. Consistent with these observations, a previous study confirmed that CaO derived from duck eggshells is highly sensitive to humidity. It rapidly converts to Portlandite (Ca(OH)_2_) upon removal from the desiccator [88,89,90]. In the present study, partial rehydration may have occurred during sample handling or characterization under ambient laboratory conditions following calcination. The formation of calcium hydroxide leads to compositional changes in the calcined material. This may affect its physicochemical properties. Therefore, rigorous moisture control may be necessary in industrial applications to limit the formation of calcium hydroxide (Ca(OH)_2_) and to obtain the desired final products.

The purity of CaO can be maintained by storing the material in an airtight container under an inert atmosphere, such as nitrogen. Alternatively, a desiccator with a reliable sealing system can minimize exposure to ambient humidity. In addition, incorporating effective desiccants, such as silica gel, molecular sieves, or calcium sulfate, is essential for maintaining low humidity levels [91,92]. Following these storage protocols might help prevent rehydration and the conversion of CaO to Ca(OH)_2_. To further elucidate the microstructural evolution associated with calcination, detailed SEM observations are presented in the following section.

### 3.4. SEM Results

**Figure 12:**
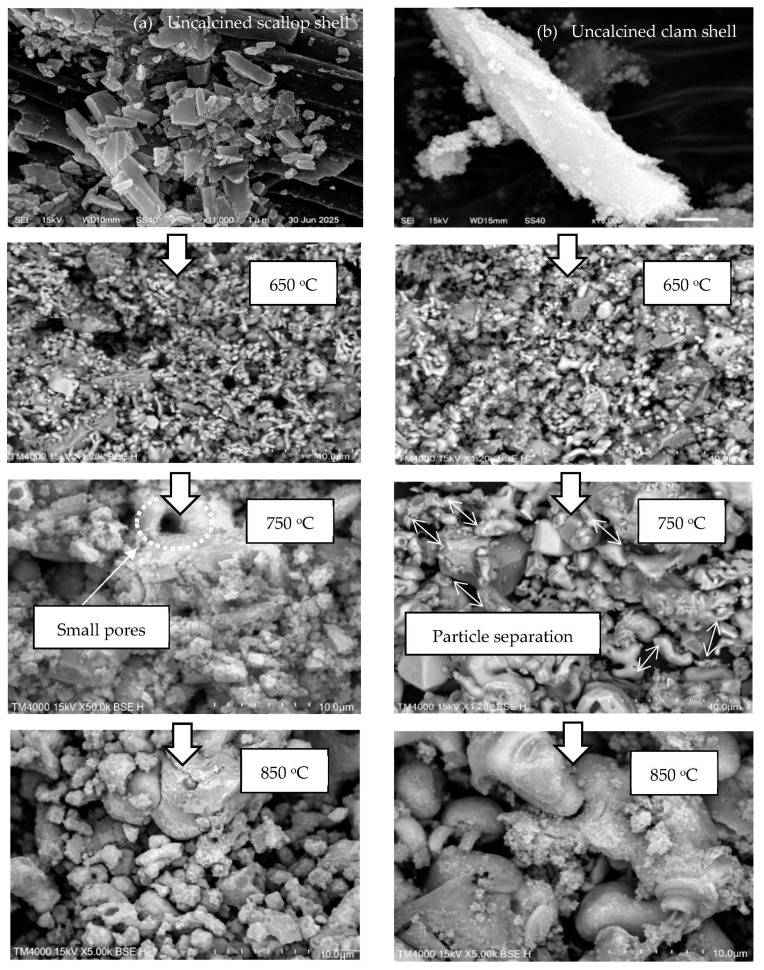
SEM micrographs of (**a**) scallop shell and (**b**) clam shell before and after calcination at 650 °C, 750 °C, and 850 °C.

Scanning electron microscopy (SEM) was employed to investigate the natural scallop and surf clam shells in both their pre- and post-calcination states, as shown in Figure 12, which reveals their morphology and internal microstructural characteristics. The SEM observations were conducted at magnifications of approximately 5.000 to 15.000 times. In the uncalcined state, scallop shell particles exhibit rhombohedral crystals, consistent with the dominance of the calcite CaCO_3_ phase [93,94,95,96]. In contrast, surf clam shell particles display needle-like and elongated morphologies, which are characteristic of the aragonite CaCO_3_ polymorph [97,98,99,100].

After calcination, small CaO particles with a non-uniform distribution were observed, accompanied by increased particle separation due to CO_2_ evolution during thermal decomposition. This heterogeneous distribution can be attributed to local temperature gradients and variations in the initial particle size during calcination [67]. Moreover, CO_2_ release promotes interparticle separation and pore formation, resulting in an irregular distribution of CaO particles [101]. At 650 °C and 750 °C, the SEM images show that the materials undergo progressive microstructural collapse, developing granular surface textures and pores indicative of advanced CaCO_3_ destabilization to CaO, as corroborated by XRD and TG-DTA analyses.

Furthermore, the observed structural transformation is attributed to the thermal degradation of organic constituents and the release of bound water during high-temperature treatment, resulting in the formation of a porous surface structure [102]. Meanwhile, at the highest temperature (850 °C), both materials exhibit fully transformed morphologies dominated by porous, granular CaO structures with extensive interparticle voids. The disappearance of the CaCO_3_ structure, together with the formation of a highly porous CaO phase, may suggest near-complete thermal decomposition.

In addition, to highlight comparison among the analytical techniques, Table 4 below summarizes the phase evolution of scallop and surf clam shells during calcination, as identified by solid-state ^13^C MAS NMR, XRD, TG-DTA, and SEM. Table 4 highlights the progressive transformation of calcium carbonate polymorphs and the formation of CaO as the calcination temperature increases, culminating in essentially complete decarbonation at 850 °C.

## 4. Prospects for Cementitious Applications

Biowaste materials considered for use as supplementary cementitious materials (SCMs) are typically divided into two categories: organic materials, such as agricultural and forestry residues, and inorganic materials, including marine-derived seashells [103]. It has been confirmed that the CaO amount depends on the origin, shell type, and calcination temperature, which is around 500–1000 °C. Researchers have also recommended several essential processing steps prior to the utilization of seashell waste, including cleaning, calcination, and grinding to the desired particle size. In particular, the washing process reduces impurities and salt content, specifically chloride ions. Calcination of seashell-based biowaste induces thermal decomposition, resulting in a composition similar to ordinary Portland cement (OPC), which is dominated by high CaO content [104,105]. After appropriate grinding, calcined products can be effectively employed as SCMs. Compared with conventional waste-disposal practices, valorizing seashell biowaste as SCMs represents a sustainable, low-carbon strategy that simultaneously reduces waste and partially substitutes Portland cement [106,107].

The physicochemical characterization conducted in the present study may directly reflect the potential SCM behavior of the investigated materials. XRD analysis confirmed that the raw seashell powders are dominated by crystalline CaCO_3_ phases, specifically calcite in scallop shells and aragonite in surf clam shells. Following calcination, solid-state ^13^C NMR and XRD results collectively confirmed the transformation of CaCO_3_ to CaO, yielding a high-calcium oxide material chemically comparable to that of OPC clinker. This transformation is essential because CaO may be the primary reactive phase governing early hydration behavior and strength development in cementitious systems. Further, CaO from calcined shells may participate in cement hydration thereby enhancing the formation of C-S-H and other hydration products through reactions with clinker phases [108]. The incorporation of seashell-derived CaO may induce rapid hydration, thereby markedly accelerating cement hydration kinetics.

Moreover, the increased reactivity facilitates the generation of key hydration products, notably C-S-H and ettringite, thus enhancing early-age strength and contributing to significant microstructural densification through the formation of additional C-S-H gel [109,110]. The present study shows that thermal treatment of seashell biowaste at an elevated temperature (850 °C) results in the complete decomposition of CaCO_3_, which may produce highly reactive CaO, as verified by solid-state ^13^C NMR, XRD, and TG-DTA analyses. Upon hydration, this thermally activated CaO may further promote C-S-H formation and contribute to matrix densification. These results may indicate that incorporating a small amount of thermally treated seashell powder enhances concrete performance by accelerating hydration and increasing C-S-H formation. The experimental findings of this study, including the high CaCO_3_ content of the raw powders, the confirmed CaCO_3_-to-CaO transformation upon calcination, and the particle morphology observed by SEM, provide a robust physicochemical basis for assessing their potential as a source of reactive CaO in future investigations. Although mechanical performance was not evaluated, the findings indicate possible applications and replacement levels when considered alongside existing literature.

Moreover, compressive strength is commonly used as an indicator of concrete’s mechanical performance [111]. It reflects the ability of concrete material to withstand applied loads without failure. An increase in compressive strength indicates improved load-bearing capacity, thereby enhancing structural stability and long-term durability of the concrete. As presented in Table 5, recent investigations indicate that CaCO_3_ can serve as a partial substitute for aggregates or cement, while maintaining or improving the compressive and flexural strength of concrete. The optimal incorporation level of seashell materials in concrete may depend on the type of substitution applied. Typically ranging from 5 to 20% for fine aggregates, 3–10% for coarse aggregates, and 5–8% for cement substitution [112,113,114].

At low replacement levels (≤10 wt%), seashell powder generally maintains or only slightly reduces compressive strength. For instance, a 5% replacement with calcined seashell powder achieved 83.69% of the control strength, while the uncalcined counterpart reached 92.32% at the same dosage [25]. In contrast, another study reported that a 5% replacement resulted in a maximum compressive strength of 112.91 MPa at 56 days [115]. However, increasing the replacement level to 10% might lead to noticeable strength reductions, with compressive strength decreasing to 78.27% and 80.07% of the control for calcined and uncalcined powders, respectively [25]. The observed decline can mainly be ascribed to interference with the cement hydration process, an increase in pore volume, and the development of a less dense, more heterogeneous microstructure [112,113,114,115,116].

A similar trend is observed in flexural performance; at low concentrations (≤10 wt%), they exhibit a positive trend. Replacements of 5% and 10% using calcined seashell powder resulted in flexural strengths of 104.31% and 104.04% relative to the control mixture [25]. In contrast, higher replacement levels (>10 wt%) significantly affected mechanical performance. A 25% replacement with waste oyster shell powder (WOSP) has been reported to cause a significant reduction in compressive strength, even at 12% replacement [117]. These findings suggest that although limited seashell incorporation can provide filler and nucleation effects, excessive substitution may result in cement dilution, higher porosity, and a deterioration of matrix continuity [118,119,120].

**Figure 13:**
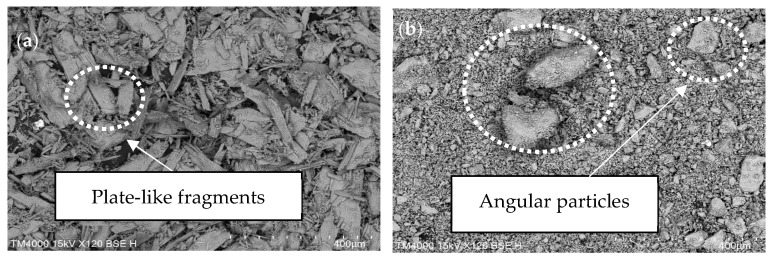
SEM images of ground uncalcined (**a**) scallop shell and (**b**) clam shell powders. The scallop shell exhibits elongated, plate-like fragments, while the clam shell shows angular particles. Both materials exhibit irregular, rough morphologies that may increase interparticle friction and water demand in cement or composite materials.

These mechanical trends appear to be associated with the physicochemical characteristics identified in this study. XRD, solid-state ^13^C NMR, and SEM analyses confirm that the shell-derived material consists predominantly of CaCO_3_, which transforms into CaO upon calcination. At moderate levels, CaO may enhance hydration by forming Ca(OH)_2_, thereby promoting continued cement reactions and microstructural densification. However, excessive CaO may induce adverse effects, including expansion, increased porosity, and microstructural instability. SEM images of the ground seashell powders (Figure 13) reveal distinct morphological characteristics between the two materials. The scallop shell displays elongated, plate-like fragments, while the clam shell consists of more angular particles. Despite these differences in particle shape, both materials share irregular, rough surface textures. These morphological features are significant from a mixed-design perspective, as rough, angular particles may increase interparticle friction and increase water demand in cementitious systems. Consequently, incorporating these seashell powders may reduce the overall workability of the mix, which should be considered when optimizing the water-to-binder ratio. In summary, seashells used at low replacement levels may help maintain or slightly improve compressive strength. However, higher dosages tend to reduce strength due to increased porosity and microstructural weakening. Therefore, careful control of seashell or CaO content is necessary to achieve balanced performance and durability in concrete.

**Table 5:** Research on the application of seashell-derived biogenic CaCO_3_ as a partial cement replacement.

Biogenic Source	Preparation Method	Cement or Aggregate Replacement Level (wt%)	Results			Ref.
			Compressive Strength (28 Days) (MPa)	Flexural Strength (MPa)	Microstructure	
Clams	Oven dry at 30 min at 100 °C, Uncalcined	5%	26.0–26.3	15.5–15.6	Slightly improved workability and no significant increase in porosity.	[25]
	Calcination at 800 °C at 5 h	20%	22.0–22.5	15.0–15.6	Not included	
		5%	17.7–18.3	12.4–12.9	Partial CaCO_3_ to CaO increased porosity and weakened matrix cohesion.	
		20%	16.6–18.1	12.1–13.2	More pronounced porosity promoted microcrack initiation and weakened the particle matrix interface	
*Senilia senilis*	Shells were cleaned, air-dried to constant mass, crushed using a Los Angeles machine, and sieved to obtain particles between 4.75 and 12.5 mm.	10% (coarse aggregate replacement)	23.13	Not reported	A slight reduction in strength was observed; however, the results remain within the acceptable range for M20-grade concrete.	[121]
		50%	14.42	Not reported	Significant strength loss; unsuitable for structural grade.	
Gastropod seashell	Oven-dried at 45 °C for 24 h, hammer-crushed, and ball-milled for 1 h (20 steel balls, 2.5 kg batch) to a median particle size of ~228 µm, then used for extrusion-based 3D printing.	Fine aggregate (river sand) replacement: 0% (SS0)	X:35.59 Y:41.67 Z: 39.02	9.67	Denser matrix, lower density 5%	[21]
		15 wt% sand replacement (SS15)	X:16.29 Y:17.43 Z: 15.57	7.34	Porosity increased by around 20%	
		30 wt% sand replacement (SS30)	X:16.11 Y:18.12 Z: 16.24	4.50	Porosity increased by around 60%	
Oyster shell	Shells were washed to remove impurities, oven-dried (~105 °C), crushed, ground, and sieved into fine powder prior to mixing.	10%	32.18	4.55	Denser and more compact microstructure than the control. Increased formation of secondary C-S-H and C-A-S-H gels due to pozzolanic reaction.	[122]
		15%	26.73	3.94	Noticeable increase in micro-pores and voids. SEM images showed a less continuous C-S-H network.	
Scallop shell	The shells were washed, oven-dried at 105 °C for 24 h, ball-milled, and sieved; particles 63–125 µm were used as filler, and particles smaller than 63 µm were used as cement replacement.	0% (Control)	51.57	Not reported	The microstructure exhibited a porous matrix with visible microcracks.	[123]
		15% filler only (S0F15)	51.74 (~0% change)	Not reported	The microstructure exhibited a denser matrix, reduced pore volume, and enhanced C-S-H formation.	
		15% replacement + 15% filler (30% total) (S15F15)	44.08 (~15% reduction)	Not reported	Increased calcite; reduced portlandite; compact ITZ.	
		30% replacement + 15% filler (45% total) (S30F15)	38.91 (~25% reduction)	Not reported	Highest calcite (~25%), lower CH, dense matrix, but dilution effect observed.	
Peruvian scallop	Washed manually, sun-dried, crushed, and sieved (1.19–4.75 mm), with particles smaller than 1 mm discarded.	5–20% replacement	Slight increase at 28 days (~5–10%)	Not reported	More compact microstructure with enhanced particle interlocking.	[112]
		40% replacement	Slight reduction (<10%)	Not reported	Angular shell particles promote mechanical interlocking.	
		60% replacement	Pronounced decline in strength	Not reported	Excessive void formation.	
Cockle shell	The shells were washed to remove impurities, oven-dried at 105 ± 5 °C for 24 h, crushed, and finally sieved to obtain particles smaller than 1.18 mm.	5–15%	Compressive strength increased up to 10% cockle shell replacement, reaching 29.75 MPa, but decreased below the control at 15%	Not reported	At 10%, improved particle packing and balanced porosity enhanced strength; at 15%, increased porosity and water absorption reduced strength.	[124]

## 5. Conclusions

This study focuses on the thermal evolution and phase transformation of scallop and surf clam shells during calcination. Although the results provide valuable insights into the decomposition behavior of biogenic calcium carbonate and the formation of CaO, the practical performance of the calcined materials in cementitious systems was not evaluated. Specifically, cement hydration behavior, surface area, pozzolanic activity, mechanical properties, durability, and compatibility with cement-based mixtures were not investigated. Therefore, the proposed use of calcined seashells as partial cement replacement materials should be regarded as a promising future application that requires verification through comprehensive evaluations of cement hydration, surface area, mechanical performance, and durability. Moreover, although CaO formation was successfully confirmed after calcination, the free CaO content and its potential effects on hydration behavior, expansion, and volume stability were not evaluated in the present study. Therefore, continued investigations are necessary to assess the suitability of the calcined shell materials for practical cementitious applications.

In this study, solid-state ^13^C NMR spectroscopy clearly distinguished calcite and aragonite phases in uncalcined shells and enabled direct monitoring of the attenuation of the carbonate signal during calcination. Scallop shells, predominantly composed of calcite, exhibited gradual carbonate signal reduction with increasing temperature, whereas clam shells, rich in aragonite, underwent a transformation from aragonite to calcite prior to complete decarbonation. At 850 °C, the disappearance of all carbonates related to ^13^C resonances confirmed full decomposition of CaCO_3_ and the formation of CaO, consistent with XRD phase identification and TG-DTA mass loss analysis. These results underscore the potential of solid-state ^13^C NMR as a valuable tool for analyzing CaCO_3_ decomposition processes. While the present ^13^C MAS NMR analysis provided qualitative information on the evolution of carbonate species during calcination, the quantitative determination of residual carbonate phases was beyond the scope of this study. The available NMR dataset was primarily acquired for qualitative phase identification and was not optimized for rigorous quantitative analysis. Therefore, the NMR results were interpreted qualitatively and used in conjunction with XRD, TG-DTA, and SEM findings. Future investigations employing quantitative NMR approaches may provide further insight into the quantitative assessment of residual carbonate species.

Moreover, TG-DTA thermal analysis revealed three distinct mass-loss stages: initial moisture evaporation below 125 °C, decomposition of organic components between 125 and 550 °C, and major CaCO_3_ decarbonation between 550 and 800 °C. SEM observations further confirmed the corresponding microstructural evolution, demonstrating a transition from dense crystalline carbonate morphologies to highly porous CaO structures resulting from CO_2_ release during calcination. Furthermore, the presence of a Ca(OH)_2_-associated thermal peak in the TG-DTA profile suggests partial hydration of CaO as a result of exposure to ambient moisture. This finding underscores the necessity for proper storage conditions to prevent unintended phase transformations. Incorporating low-level seashell-derived materials as partial cement replacement generally maintains adequate compressive and flexural strength, while potentially enhancing microstructural densification through filler effects and improved particle packing. However, higher replacement levels (>15–20 wt%) may lead to increased porosity, weakened matrix cohesion, and consequently reduced mechanical strength and durability.

Despite the promising environmental and mechanical advantages of incorporating seashells into concrete, several critical aspects remain insufficiently understood and require further detailed investigation, such as:

1. Conventional approaches have predominantly relied on calcination to enhance reactivity, despite their substantial energy demand and associated CO_2_ emissions. Non-calcined or low-energy activation approaches may offer a more sustainable path forward, and the development of these alternatives has become increasingly critical.
2. Several studies report that calcined seashell powder, predominantly composed of CaO, can alter hydration kinetics and prolong the setting time of cement paste. However, the mechanisms governing this behavior might remain unclear. More comprehensive investigations are required to systematically evaluate the influence of CaO-rich seashell powder on early hydration reactions, setting characteristics, and microstructural development.
3. A detailed economic and environmental evaluation is needed to determine whether the energy used for calcination can be compensated for by reducing clinker production, to ensure both sustainability and cost-effectiveness in practical applications.

## Figures and Tables

**Table 1:** Results of the NMR data of the CaCO_3_ and OPC-limestone mix phases, adopted from [28,34].

Phase	Calcite	Aragonite	Vaterite	References
^13^C chemical shift (ppm)	168.80	171.20	168.70	[28]
	169.00	-	-	[28]
	168.70	-	-	[28,45]
	168.21	170.49	170.12	[34]
	-	-	169.07	[34]

**Table 2:** Solid-state ^13^C NMR chemical shifts reflecting the evolution of CaCO_3_ polymorphs in scallop and clam shells before and after calcination at varying temperatures.

Compound	Uncalcined	Calcination Temperatures		
		650 °C	750 °C	850 °C
	(ppm)	(ppm)	(ppm)	(ppm)
Scallop shells				
CaCO_3_-calcite	168.7 (signal detected)	168.7 (signal detected)	- (signal decayed)	- (signal decayed)
CaCO_3_-aragonite	-	-	-	-
CaCO_3_-vaterite	-	-	-	-
Surf clam shells				
CaCO_3_-calcite	-	168.7 (signal detected)	168.7 (residual signal)	- (signal decayed)
CaCO_3_-aragonite	171.2 (signal detected)	- (signal decayed)	- (signal decayed)	- (signal decayed)
CaCO_3_-vaterite	-	-	-	-

**Table 3:** Weight-loss stages of uncalcined samples determined by TGA.

Raw Sample	Weight Loss			
	Moisture (%)	Organic Matter (%)	CO_2_ (%)	Total (%)
Scallop shell	0.10	1.80	46.21	48.20
Clam shell	0.15	1.85	43.15	46.00

**Table 4:** Comparative summary of phase evolution in scallop and surf clam shells observed by different analytical techniques during calcination.

No	Temperature	^13^C MAS NMR	XRD	TG-DTA	SEM
1	Unheated	Scallop: dominant calcite signal (~168 ppm). Surf clam: dominant aragonite signal (~171 ppm).	Scallop: calcite dominant. Surf clam: aragonite dominant.	Stable CaCO_3_ phases.	Dense shell microstructure with intact morphology.
2	650 °C	Scallop: reduced calcite signal intensity. Surf clam: aragonite signal decreases; weak signal detected.	Scallop: initial CaO formation with residual calcite. Surf clam: aragonite decreases, calcite and CaO phases appear.	Initial CO_2_ release is associated with the onset of CaCO_3_ decomposition.	Microstructural changes were observed.
3	750 °C	Significant reduction in carbonate signals in both shells. Surf clam: transformation to calcite is largely completed before decomposition.	Increased CaO content and reduced carbonate phases in both shells.	Significant CO_2_ release and major mass loss due to CaCO_3_ decomposition.	Progressive microstructural degradation and porosity were observed.
4	850 °C	Carbonate signals disappear in both shells, indicating essentially complete decarbonation.	CaO becomes the dominant phase in both shells; residual carbonate phases are negligible or absent.	CO_2_ released is completed, indicating essentially complete decarbonation.	An extensive, porous, and fragmented microstructure was observed

## Data Availability

The original contributions presented in this study are included in the article. For further inquiries, please contact the corresponding author.
